# Investigating the Prevalence of Hypercalciuria in Children Aged 2–16 Years With Asymptomatic Microscopic Hematuria in 2020 in Tehran Children's Medical Center

**DOI:** 10.1002/ccr3.9575

**Published:** 2024-11-13

**Authors:** Izat MohammadKhawajah, Sima Shamshiri Khamene, Amir Ali Mahboobipour, Elahe Radmehr, Mastaneh Moghtaderi

**Affiliations:** ^1^ Tehran Heart Center Tehran University of Medical Sciences (TUMS) Tehran Iran; ^2^ Tracheal Diseases Research Center, National Research Institute of Tuberculosis and Lung Diseases Shahid Beheshti University of Medical Sciences Tehran Iran; ^3^ Colorectal Research Center, Imam Khomeini Hospital Complex Tehran University of Medical Sciences (TUMS) Tehran Iran; ^4^ Universal Scientific Education and Research Network (USERN) Tehran Iran; ^5^ Pediatric Chronic Kidney Diseases Research Center & Gene, Cell, and Tissue Research Institute, Children's Medical Center Tehran University of Medical Sciences (TUMS) Tehran Iran

**Keywords:** hypercalciuria, kidney stones, microscopic hematuria, prevalence

## Abstract

The prevalence of hypercalciuria in children is 3%–10% globally and up to 35% in the United States. Hypercalciuria in children has many presentations; it causes different metabolic disorders and can negatively affect a child's growth. It also increases the risk of low bone mineral density and urinary tract infections. In addition, it is the most widespread cause of persistent microscopic hematuria. Changes in the diet and medications in cases of advanced stage can be useful to prevent further complications. This study aimed to determine the prevalence of hypercalciuria and to investigate its relationship with different factors in children aged 2–16 years with asymptomatic microscopic hematuria in 2020 in the Children's Medical Center hospital. This retrospective cross‐sectional study was performed in a population of children aged 2–16 years old with asymptomatic microscopic hematuria who were referred to the Children's Medical Center clinic in 2020. Data such as age, sex, serum creatinine level, and proteinuria were extracted from the patient's medical records, and their relationship with hypercalciuria was analyzed using logistic regression analysis. According to the inclusion criteria, 166 children with asymptomatic microscopic hematuria (72 boys and 94 girls) were included in this study. The prevalence of hypercalciuria (ratio of random urine calcium to creatinine more than 0.2) in these patients was estimated at 25% with a confidence interval of 18%–32%. In order of prevalence, the most common conditions accompanying microscopic hematuria were kidney stones, urinary tract infections, and proteinuria. The age of patients with hypercalciuria was 2 years younger on average. Each year of age increase and every 5 years of age increase between the ages of 2 and 16 years reduced the chance of hypercalciuria in this category of patients by 12% and 45%, respectively. Our findings also showed that children with a positive history of kidney stones were about 2.2 times more likely to have hypercalciuria than their counterparts, which is considered a medium effect size. Our results showed that hypercalciuria in children with hematuria is significantly related to younger age and a positive history of kidney stones.

**Summary**

- About 25% of children with asymptomatic microscopic hematuria have hypercalciuria.
- Hypercalciuria in hematuric children with hematuria is significantly related to younger age and a positive history of kidney stones.
- Each year of age increase and every 5 years of age increase, reduces the chance of hypercalciuria by 12% and 45%, respectively.

## 1 Introduction

Idiopathic hypercalciuria is a situation in which the elimination of calcium increases in the absence of hypercalcemia or other causes of hypercalciuria [1, 2]. Hypercalciuria is defined as a urine calcium/creatinine ratio of more than 0.2 [3]. It is the most frequently recognized metabolic risk factor for calcium nephrolithiasis [4, 5, 6], which may lead to various urinary symptoms with noncharacteristic ones including abdominal pain, erythrocyturia, hematuria, dysuria, and enuresis [7, 8, 9]. Twenty‐four‐hour urine analysis is the best way to diagnose hypercalciuria [10, 11].

The prevalence of hypercalciuria in children is up to 35% in the United States and 3%–10% globally. It is also the most widespread cause of persistent microscopic hematuria [3, 12, 13]. Microscopic hematuria generally means hematuria that can be detected only by microscopy or urine dipstick [14]. It is defined as more than five erythrocytes per high‐power field (HPF) on microscopic evaluation of urinary specimens or as some other references say more than three erythrocytes per HPF.

Hypercalciuria is mostly benign [13], but some of its complications are as follows: Hypercalciuria reduces bone mineral density, and as the peak bone mass formation starts from childhood, any disturbance in this process can result in low bone density [15, 16, 17]. Hypercalciuria increases the risk of urinary tract infections by causing microscopic hematuria, higher Na excretion, and injury to the uroepithelium [18, 19, 20, 21, 22]. Also, a relationship between idiopathic hypercalciuria and persistent hematuria has been shown [23].

This study aimed to determine the prevalence of hypercalciuria in children aged 2–16 years with asymptomatic microscopic hematuria in 2020 in the Children's Medical Center hospital.

## 2 Materials and Methods

In this retrospective cross‐sectional study, all the children who were referred to the nephrology clinic of the Children's Medical Center in 2020 with asymptomatic microscopic hematuria were investigated. Patients included were 2–16 years old.

According to the hospital protocol and reference literature, the calcium and creatinine levels in random or 24‐h‐urine samples, and sometimes the serum levels of calcium, creatinine, magnesium, and uric acid were measured. Also, when necessary, imaging was used to identify the stones. All of this information was recorded in the patient's history sheet during their visits to the clinic; these files were transferred to the hospital's archive afterward and used again for follow‐up on each visit. The patients' characteristics including age, sex, treatment start date, response to the treatment, as well as the level of patient adherence to their diet, were gathered from the files and documented in the datasheet. Finally, the extracted information was entered into SPSS‐20 software. It should be noted that patients whose medical records lacked detail were not included in this study.

All the data were coded in SPSS‐20 software; if the analyses could not be done with SPSS (especially the prevalence variable confidence interval [CI] determination), they were done with STATA‐14 software. At first, descriptive statistics (percentage for qualitative variables and mean and standard deviation or median and interquartile range [IQR] for quantitative variables) were calculated. Then, according to the type of independent variables (qualitative or quantitative), if the assumptions of the binary logistic regression statistical analysis and the effect sizes of this test were met, we checked the risk factors or predictors. When the assumptions were not established, we tried to establish them using methods such as variable change. As much as possible, we did not go for nonparametric tests because of statistical power. Finally, by using multivariate analysis, the final relationship of each of these factors with the outcomes was investigated.

## 3 Results

According to the inclusion criteria, 166 patients with asymptomatic microscopic hematuria, including 72 boys and 94 girls, were included in this study and analyzed. Demographic characteristics and underlying diseases are summarized in Table 1. Eighteen percent of the investigated children were adolescents (children over 10 years old), and 82% were in their childhood period (10 years old or younger). The median (IQR) age of the total study population and the children with hypercalciuria was 6 (5–10) years and 5 (3–8) years, respectively.

**TABLE 1:** Demographic characteristics and underlying diseases in the studied population.

Variable	Group without hypercalciuria, numbers = 125 (75%)			Group with hypercalciuria, numbers = 41 (25%)			Total numbers = 166 (100%)		
	Range	Median	IQR	Range	Median	IQR	Range	Median	IQR
Age (year)	2–16	7	5–10	2–16	5	3–8	2–16	6	5–10
Serum creatinine (mg/dL)	0.15–2.9	0.6	0.5–0.7	0.1–0.7	0.54	0.5–0.6	0.1–2.9	0.6	0.5–0.7
	Frequency (*N*, %)			Frequency (*N*, %)			Frequency (*N*, %)		
Male	51 (40.8)			21 (51.2)			72 (43.4)		
Age > 10 years (adolescent)	24 (19.2)			6 (14.6)			30 (18.1)		
Underlying disease									
Urolithiasis	33 (26.4)			20 (48.8)			53 (32.1)		
Proteinuria	18 (14.4)			4 (9.8)			22 (13.2)		
UTI	12 (9.6)			7 (17.1)			19 (11.4)		
Family history of kidney disease	7 (5.6)			4 (9.8)			11 (6.6)		
Nocturia	6 (4.8)			1 (2.4)			7 (4.2)		
VUR	6 (4.8)			0 (0)			6 (3.6)		
FTT	1 (0.8)			2 (4.9)			3 (1.9)		
RTA distal	2 (1.6)			1 (2.4)			3 (1.9)		

Abbreviations: FTT, failure to thrive; RTA, renal tubular acidosis; UTI, urinary tract infection; VUR, vesicoureteral reflux.

**FIGURE 1:**
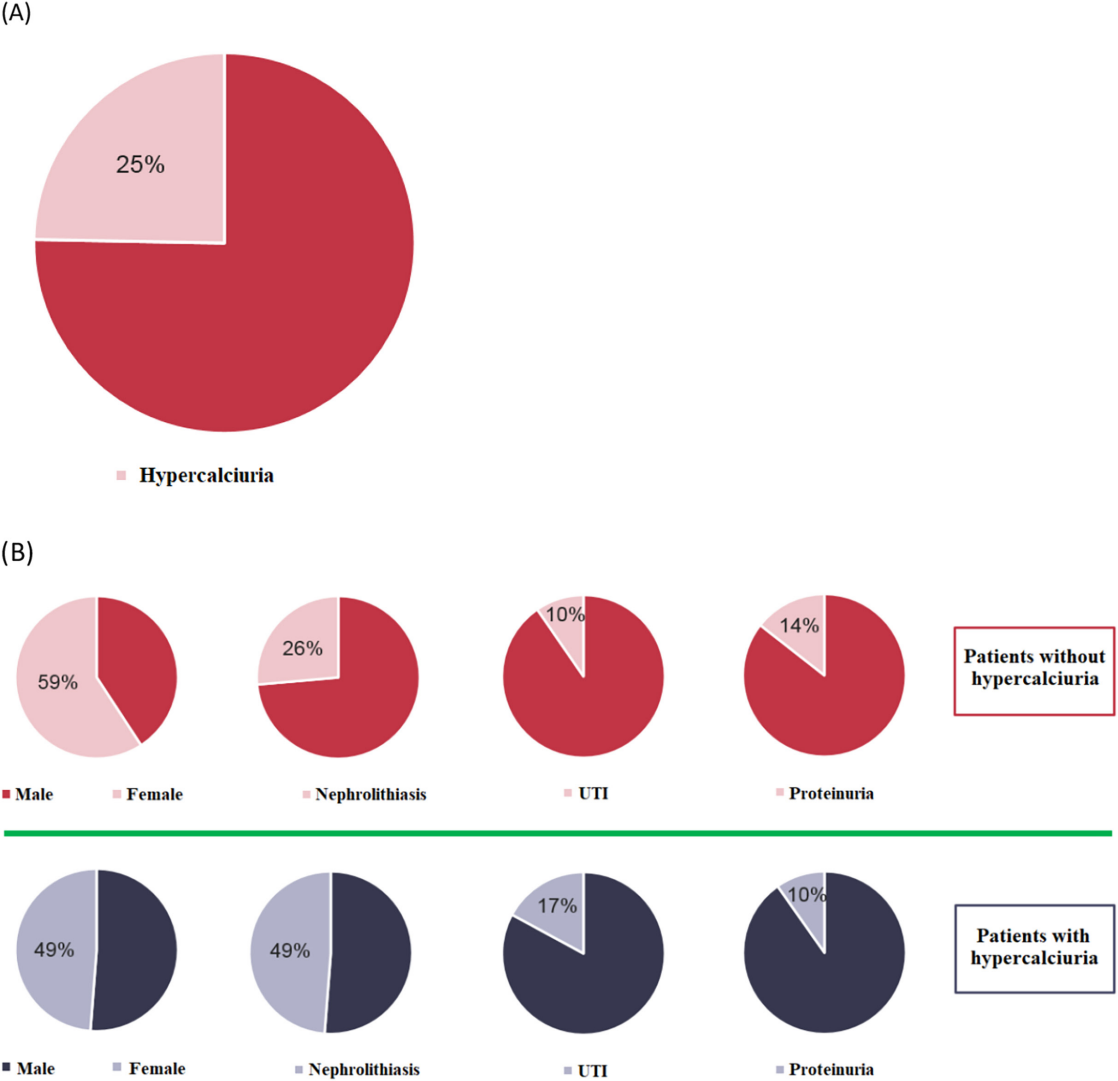
(A) Pie chart of prevalence of hypercalciuria in patients with microscopic hematuria. (B) Comparative pie chart of demographic features and accompanying cases between two groups. UTI: urinary tract infection.

In this study, the prevalence of hypercalciuria (ratio of random urine calcium to creatinine more than 0.2) in patients with asymptomatic microscopic hematuria was estimated at 25% with a CI of 18%–32% (Figure 1A).

Family history of renal problems in first‐degree relatives of the total study population was 6.6%. In order of prevalence, the most common conditions associated with microscopic hematuria were a positive history of kidney stones (before or during the approach to hematuria), urinary infection (before or during the approach to hematuria), and proteinuria (before or during the approach to hematuria) (Figure 1B).

**FIGURE 2:**
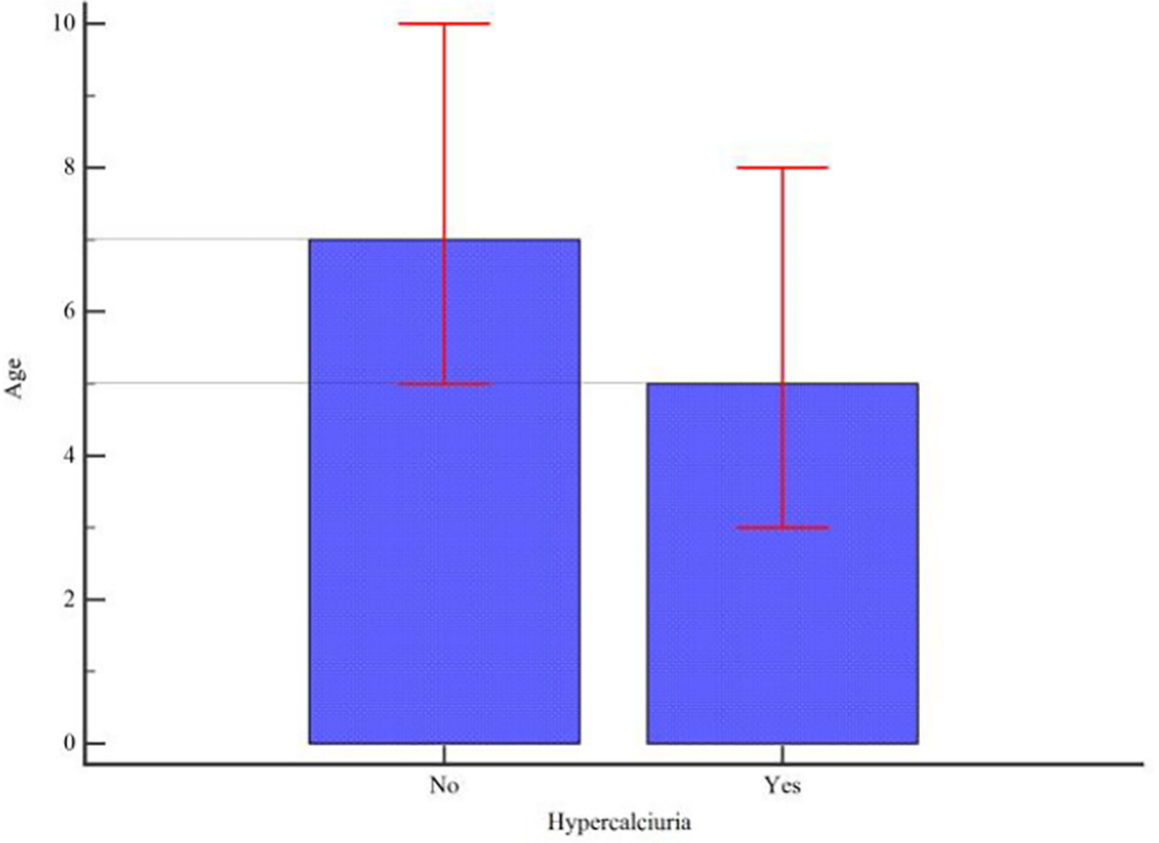
Bar chart of age (years) in the two groups (error bar represents interquartile range [IQR]).

The prevalence of kidney stones in patients with hypercalciuria was about 20% higher than in patients without hypercalciuria. Also, the age of patients who had hypercalciuria was 2 years younger on average (Figure 2).

To investigate the risk factors of hypercalciuria in patients with microscopic hematuria, logistic regression was used to calculate the *p* value and OR effect size. Univariate analyses showed that age and history of kidney stones are related to hypercalciuria (*p* < 0.05). However, sex, serum creatinine level, history of proteinuria, history of urinary tract infection, and family history of kidney disease in first‐degree relatives were not related to hypercalciuria (*p* > 0.05). Multivariate analysis was used for age and history of kidney stones to correct their effect size (Table 2). Both of these factors had a significant relationship with hypercalciuria in the studied patients (*p* < 0.05), with adjusted OR = 0.88 (95% CI: 0.79–0.99) and adjusted OR = 2.19 (95% CI: 1.02–4.69) for age and history of kidney stones, respectively (Table 2).

**TABLE 2:** The relationship of independent variables with the presence of hypercalciuria in patients with microscopic hematuria (*p* is based on the logistic regression test).

Variables	OR (95% CI)	*p*
**Age (for every year of age)	0.86 (0.76–0.96) Adjusted: 0.88 (0.79–0.99)	0.009 Adjusted: 0.033
Serum creatinine level	0.08 (0.01–1.04)	0.054
Sex (female to male)	0.66 (0.32–1.33)	0.244
Family history of kidney disease	1.82 (0.51–6.57)	0.359
Proteinuria	0.64 (0.20–2.02)	0.450
**Urolithiasis	2.66 (1.28–5.51) Adjusted: 2.19 (1.02–4.69)	0.009 Adjusted: 0.043
UTI	1.94 (0.71–5.31)	0.198

Abbreviations: CI, confidence interval; OR, odds ratio; UTI, urinary tract infection.

** *p* < 0.05.

## 4 Discussion

The main goal of this study was to investigate the prevalence of hypercalciuria in children with asymptomatic microscopic hematuria aged 2–16 years who were referred to Tehran Children's Medical Center in 2020. In order of prevalence, we found that the most common conditions associated with microscopic hematuria were a positive history of kidney stones, urinary infection, and proteinuria. Family history of kidney problems in first‐degree relatives was 6.6% in total, which was not consequential.

In a descriptive cross‐sectional study conducted by Maryam Esteghamati and her colleagues [24] in 2014 at Bandar Abbas Children's Hospital (south of Iran) on 321 children between 2 months and 14 years old, 153 (47.7%) had idiopathic hypercalciuria, and the prevalence of idiopathic hypercalciuria in children with microscopic hematuria was 54.9%, whereas in our study, the prevalence of hypercalciuria in patients with asymptomatic microscopic hematuria was estimated at 25% (CI: 18–32), which is lower. Also, the prevalence of hypercalciuria in children with kidney stones confirmed by ultrasound was 49.1% in Esteghamati's study [24], whereas the prevalence of hypercalciuria in children with microscopic hematuria with a positive history of kidney stones was 37.7% (20 out of 53) in our study.

In a study by Valavi and colleagues [25] in 2019, about 522 children with hematuria were enrolled in a prospective cross‐sectional study. Eighty‐eight‐point‐5% had microscopic hematuria with most of them being mild hematuria. No statistical relationship was found between hematuria and metabolic disorders in urine, including proteinuria. In line with their findings, we showed that hypercalciuria in children with hematuria is not related to proteinuria.

Ertan and colleagues [26] retrospectively reviewed the records of 85 children with kidney stones referred to the Pediatric Nephrology Department of Celal Bayar University Manisa between 2004 and 2010. In patients who were < 12 months old at the time of diagnosis, hypercalciuria was the most common urinary metabolic risk factor.

In our study, the prevalence of kidney stones in patients with hypercalciuria was about 20% higher than in patients without hypercalciuria, which indicates a significant relationship between kidney stones and hypercalciuria in asymptomatic microscopic hematuria. Also, the age of patients who had hypercalciuria was 2 years younger on average. Both age and history of kidney stones were significantly related to hypercalciuria in our multivariate analysis. We found that each year of age increase between the ages of 2 and 16 years reduces the chance of hypercalciuria in this category of patients by 12% (hypercalciuria is associated with younger age) and for every 5 years of age increase, the chance of hypercalciuria decreases by 45%. Concerning kidney stones, our findings showed that people with a positive history of kidney stones are about 2.2 times more likely to have hypercalciuria than their counterparts, which is considered a medium effect size. Therefore, in addition to being significant, the relation of both factors to hypercalciuria is not weak.

Our study had some limitations. First, as 24‐h urinalysis had not been performed in our study population, we were able to investigate hypercalciuria just by the value of the urine Ca/Cr ratio. Also, it should be noted that not all of the children with asymptomatic microscopic hematuria present to the clinic because of the nature of this condition, and so, not all of them are diagnosed. Hence, our study failed to analyze and didn't include the population of children with asymptomatic microscopic hematuria who did not come to the clinic and didn't take a U/A test. Although this study provides insight into the prevalence of hypercalciuria among the children presented to the Children's Medical Center in Tehran, caution should be exercised when generalizing these findings to other populations. Given that this is a single‐center study, its findings may not be universally applicable because of several factors, including population genetic variability, ethnic diversity, and differences in dietary habits and lifestyle.

## 5 Conclusion

In summary, the prevalence of hypercalciuria in patients with asymptomatic microscopic hematuria was 25% in our study, and the two risk factors related to the occurrence of hypercalciuria in these patients were younger age and a positive history of kidney stones.

We recommend that future studies investigate hypercalciuria in children with asymptomatic microscopic hematuria, at a larger scale such as the whole city or country, with a greater sample size to better understand the global landscape of hypercalciuria and its associated factors. Also, it would be wise to use the 24‐h urinalysis for the detection of hypercalciuria as it is the most accurate test for diagnosing hypercalciuria.

## Author Contributions

**Izat MohammadKhawajah:** conceptualization, data curation, formal analysis, investigation, methodology, project administration, writing – original draft. **Sima Shamshiri Khamene:** data curation, investigation, writing – original draft. **Amir Ali Mahboobipour:** data curation, formal analysis, methodology. **Elahe Radmehr:** writing – original draft, writing – review and editing. **Mastaneh Moghtaderi:** conceptualization, methodology, project administration, supervision.

## Ethics Statement

The study protocol was approved by the ethics committee of Tehran Children's Medical Center (reference number: IR.TUMS.CHMC.REC.1400.046).

## Consent

Written informed consent was obtained from the patients' parents or guardians to publish this report in accordance with the journal's patient consent policy.

## Conflicts of Interest

The authors declare no conflicts of interest.

## Data Availability

The data supporting this study's findings are available from the corresponding author upon reasonable request.
